# Metabolomics profiles associated with HbA1c levels in patients with type 2 diabetes

**DOI:** 10.1371/journal.pone.0224274

**Published:** 2019-11-07

**Authors:** Jun Ho Yun, Heun-Sik Lee, Ho-Yeong Yu, Yeon-Jung Kim, Hyun Jeong Jeon, Taekeun Oh, Bong-Jo Kim, Hyung Jin Choi, Jeong-Min Kim

**Affiliations:** 1 Division of Genome Research, Center for Genome Science, Korea National Institute of Health, Cheongju, Chungbuk, Republic of Korea; 2 College of Pharmacy, Chungbuk National University, Cheongju, Chungbuk, Republic of Korea; 3 Department of Internal Medicine, Chungbuk National University College of Medicine, Cheongju, Chungbuk, Republic of Korea; 4 Department of Biomedical Sciences & Department of Anatomy and Cell Biology, Wide River Institute of Immunology, Seoul National University College of Medicine, Seoul, Republic of Korea; International University of Health and Welfare, School of Medicine, JAPAN

## Abstract

Glycated hemoglobin (HbA1c) is an indicator of the average blood glucose concentration. Failing to control HbA1c levels can accelerate the development of complications in patients with diabetes. Although metabolite profiles associated with HbA1c level in diabetes patients have been characterized using different platforms, more studies using high-throughput technology will be helpful to identify additional metabolites related to diabetes. Type 2 diabetes (T2D) patients were divided into two groups based on the HbA1c level: normal (HbA1c ≤6%) and high (HbA1c ≥9%) in both discovery and replication sets. A targeted metabolomics approach was used to quantify serum metabolites and multivariate logistic regression was used to identify significant differences between groups. The concentrations of 22 metabolites differed significantly between the two groups in the discovery set. In the replication set, the levels of 21 metabolites, including 16 metabolites identified in the discovery set, differed between groups. Among these, concentrations of eleven amino acids and one phosphatidylcholine (PC), lysoPC a C16:1, were higher and four metabolites, including three PCs (PC ae C36:1, PC aa C26:0, PC aa C34:2) and hexose, were lower in the group with normal HbA1c group than in the group with high HbA1c. Metabolites with high concentrations in the normal HbA1c group, such as glycine, valine, and PCs, may contribute to reducing HbA1c levels in patients with T2D. The metabolite signatures identified in this study provide insight into the mechanisms underlying changes in HbA1c levels in T2D.

## Introduction

Rapid advances in metabolomics have enabled quantitative analyses of small-molecule metabolites in biological samples, such as blood and urine, and their associations with biological processes. The metabolic phenotype is the product of genetic and environmental factors, providing functional information reflecting the pathophysiological states of diseases [1]. Using high-throughput technology, large-scale metabolic profiling has been applied to identify metabolites involved in disease progression [2]. T2D is a multi-factorial disease; single omics approaches are insufficient for clarifying its underlying basis. Therefore, emerging metabolomics approaches have been used to complement other omics data with the goal of elucidating the mechanisms underlying the development and progression of T2D [3]. A number of studies have reported T2D-related metabolites in various cohorts, some of which have been replicated in different populations, suggesting that there are common metabolites associated with disease development and progression [4–6].

Glycated hemoglobin (HbA1c) is a form of hemoglobin chemically linked to glucose at the N-terminal end of the beta chain. It is a measure of average blood glucose levels of the previous three months and can be used as a diagnostic test of diabetic status of patients. Additionally, HbA1c is a marker for outcomes in patients with diabetes treated with drugs that lower the plasma glucose concentration. It is associated with diabetes-related complications, such as cardiovascular disease, nephropathy, and retinopathy; failing to control the level of HbA1c can accelerate the development of complications [7–9].

Chronic glycemic levels (HbA1c) are not known to fluctuate over any given period and are more stable than fasting plasma glucose, and thus are more reliable. Moreover, HbA1c levels in diabetic drug-treated patients is related to biological factors with roles in physiological homeostasis, suggesting the importance of identifying metabolites associated with HbA1c levels in patients [9, 10]. Therefore, several studies were performed to identify metabolites associated with the HbA1c levels in serum of diabetes patients. Among them, serum and urine samples from T2D patients treated with three commonly used oral antidiabetic agents for 8 weeks were analyzed to identify metabolites associated with HbA1c levels. The three metabolites, IL-8, urine citrate, and 1-methyl histidine, were identified as better indicators compared to only thiazolidinedione treatment in responders than non-responders [11]. In another recent study, the levels of two amino acids, valine and tyrosine, decreased and levels of leucine/isoleucine increased after metformin treatment in T2D patients [12]. It was reported that glucose and 1,5-anhydroglucitol were associated with change in HbA1c levels in all medication groups including metformin, sulfonylurea, and combination of metformin and sulfonylurea. Additionally, four metabolites, 2-hydroxybutanoic acid, 3-hydroxybutanoic acid, 2-hydroxypiperidine, and 4-oxoproline were identified to be associated with HbA1c decrease in metformin and combination of metformin and sulfonylurea treatments [13]. A similar study using two groups, high and normal levels of HbA1c, was also performed but serum samples were obtained from type 1 diabetes (T1D) patients, especially children and teenagers. Three metabolites, alanine, pyruvate, and branched amino acid valine, were higher in patients with high HbA1c levels and might be related to endogenous glucose production pathway from proteins as well in the case of T2D [14]. From studies that identified metabolites associated with HbA1c levels in serum of patients who did or did not undergo drug treatments, it might be speculated that the identified metabolites may vary depending on study cohorts and analysis methods. Therefore, more studies identifying metabolites related to the HbA1c levels in T2D patients will be beneficial. These metabolites could be useful biomarkers for serum HbA1c levels and act as potential targets for treatments to reduce glucose levels in T2D patients.

In this study, we produced and analyzed metabolome data of T2D patients by using targeted metabolomics approach and identified several metabolites with differences in concentrations between high and normal levels of HbA1c in T2D patients.

## Materials and methods

### Study subjects and samples

Serum samples from patients with diabetes were collected and stored at −80°C in a refrigerator at Chungbuk National University Hospital, following international ethical guidelines. All patients provided written informed consent. This study was reviewed and approved by the Chungbuk National University Hospital Institutional Review Board (Approval Number: CBNUH 2015-02-006). In total, 148 patients with a high level of HbA1c and 119 patients with a normal level were included in the discovery set. In the replication set, serum samples from 51 patients with a high level of HbA1c and 82 patients with a normal level were collected and analyzed. The discovery set was constructed to identify metabolites associated with HbA1c levels in serum of T2D patients and the replication set was constructed for validation of selected metabolites from discovery set. Gender, age, height, weight, BMI, and levels of HbA1c, glucose, and creatinine were recorded.

### Metabolomics profiling

Serum samples from patients with diabetes were analyzed by targeted metabolomics approach. To quantify metabolites, liquid chromatography (LC) and flow-injection analysis (FIA)–mass spectrometry (MS) were performed using the AbsoluteIDQ^®^ p180 Kit (BIOCRATES Life Sciences AG, Innsbruck, Austria) and quality control was performed to select metabolites for further analyses. Metabolites with significant differences in levels between the groups were identified. All procedures were performed in the same manner as previously [6, 15]. Briefly, serum samples were analyzed using the API 4000 QTRAP LC/MS/MS system (Applied Biosystems, Foster City, CA, USA) and the Agilent 1200 HPLC system (Agilent Technologies, Santa Clara, CA, USA), following the manufacturers’ instructions. Calibration standards and three different quality controls were provided with the AbsoluteIDQ^®^ p180 Kit to serve as references when calculating metabolite concentrations. Two additional pooled normal human sera samples in each plate were used as reference standards. Each metabolite had to meet the following criteria to fulfill the data quality. First, the coefficient of variation (CV) of the metabolites in a total of 10 reference standards must be below 15%. Second, 50% of the measured metabolite concentrations in both the reference and experimental samples must be above the limit of detection (LOD) that is set to three times the median value of three blank samples within each plate. After quality control processes, 122 metabolites met the following criteria and were thus selected for further statistical analysis (S1 Table).

### Statistical analyses

Statistical analyses were performed using R (version 3.0.2; http://www.r-project.org). To identify metabolites that differ in concentration between groups of patients with diabetes with high (HbA1c ≥9%) and normal (HbA1c ≤6%) levels of HbA1c in the serum, odd ratios (ORs) for single metabolites were calculated by multivariate logistic regression with 95% confidence intervals (CI). To obtain a mean of zero and a standard deviation of one, the concentration of metabolites was log-transformed and normalized. Age and sex were included as covariates. To adjust *P*-value for multiple comparisons, the false discovery rate (FDR) was calculated by using ‘p.adjust method in R package STATS’ (FDR < 0.05). The cutoff for significance was *P* < 1.0E-03 (FDR-adjusted *P*-value).

## Results

### Clinical and biochemical characteristics of the discovery and replication sets

In the discovery set, the average ages of subjects in the high HbA1c group (≥9%) and normal HbA1c group (≤6%) were 58.94 and 62.46 years, respectively. Sex and age differed significantly between the two groups. However, there were no significant differences in height, weight, BMI, and creatinine concentrations. As the two groups were divided according to HbA1c level, both HbA1c and glucose levels were significantly different between the two groups. In the replication set, the subject characteristics were similar to those in the discovery set (Table 1).

**Table 1:** Clinical characteristics of subjects in the discovery and replication sets.

Clinical and Biochemical Parameters	Discovery Set			Replication Set		
	HbA1c ≥ 9% (n = 148)	HbA1c ≤ 6% (n = 119)	P-value	HbA1c ≥ 9% (n = 51)	HbA1c ≤ 6% (n = 82)	P-value
Female (%)	41.20	30.30	<0.01	28.00	30.00	0.76
Age (year)	58.94 (11.87)	62.46 (11.82)	<0.01	56.28 (12.52)	62.48 (10.89)	<0.001
Height (cm)	162.99 (8.92)	163.93 (7.38)	0.36	166.02 (7.96)	164.03 (7.09)	0.17
Weight (kg)	66.63 (11.45)	67.09 (9.78)	0.73	69.64 (10.81)	67.17 (8.21)	0.18
BMI (kg/m^2^)	25.11 (3.97)	24.97 (2.96)	0.74	25.39 (4.12)	25.05 (2.74)	0.62
HbA1c (%)	10.36 (1.46)	5.72 (0.24)	<0.01	10.45 (1.41)	5.72 (0.25)	<0.001
Glucose (mg/dl)	214.08 (86.84)	115.08 (27.63)	<0.01	221.89 (87.0)	114.28 (24.99)	<0.001
Creatinine (mg/dl)	1.14 (0.49)	1.06 (0.33)	0.27	1.12 (0.41)	1.02 (0.33)	0.11

### Discovery of metabolites with differential concentrations between groups with high and normal levels of HbA1c

To identify metabolites with significant differences in concentrations between groups with high and normal levels of HbA1c in the discovery set, a multivariate logistic regression analysis was performed using HbA1c as a dependent variable and each metabolite as an explanatory variable, with adjustment for age and sex. The concentrations of 22 metabolites differed significantly (*P* < 1.0E-03) between the two groups. The greatest difference was observed for hexose, as expected, since glucose is a subtype of hexose (Table 2). Interestingly, many amino acid metabolites showed higher concentrations in the normal HbA1c group than in the high HbA1c group. In total, eleven amino acids (glutamine, tryptophan, histidine, lysine, valine, threonine, tyrosine, sarcosine, glycine, and serine) and two phosphatidylcholine (PC) metabolites (lysoPC a C16:1 and lysoPC a C18:0) showed significantly higher concentrations in the group with normal HbA1c than in the group with high HbA1c. However, two amino acids (citrulline and spermidine) and six PC metabolites (PC aa C34:1, PC aeC36:1, PC aa C28:1, PC aa C36:4, PC aa C26:0, and PC aa C34:2) showed significantly lower concentrations in the group with normal HbA1c than in the group with high HbA1c.

**Table 2:** Identification of metabolites that differ depending on the level of HbA1c by a multivariate logistic regression analysis in the discovery set.

Group	Metabolites	Odds Ratio (CI)	FDR-Corrected P-value
High Concentrations in the Normal HbA1c Group	Glutamine	3.02 (2.15–4.38)	1.00E-09
	Tryptophan	2.94 (2.12–4.21)	6.00E-10
	Histidine	2.65 (1.91–3.8)	3.00E-08
	Lysine	2.27 (1.68–3.17)	4.00E-07
	Valine	1.97 (1.49–2.65)	4.00E-06
	Threonine	1.96 (1.48–2.66)	7.00E-06
	Methionine	1.91 (1.44–2.59)	1.00E-05
	Tyrosine	1.70 (1.30–2.27)	2.00E-04
	lysoPC a C16:1	1.63 (1.25–2.16)	4.00E-04
	Sarcosine	1.59 (1.19–2.21)	3.00E-03
	lysoPC a C18:0	1.58 (1.22–2.09)	8.00E-04
	Glycine	1.55 (1.19–2.03)	1.00E-03
	Serine	1.52 (1.17–2.01)	2.00E-03
Low Concentrations in the Normal HbA1c Group	PC aa C34:1	0.66 (0.48–0.87)	5.00E-03
	PC ae C36:1	0.64 (0.48–0.84)	2.00E-03
	PC aa C28:1	0.64 (0.47–0.85)	3.00E-03
	Citrulline	0.58 (0.41–0.77)	6.00E-04
	Spermidine	0.57 (0.40–0.77)	6.00E-04
	PC aa C36:4	0.56 (0.40–0.75)	3.00E-04
	PC aa C26:0	0.51 (0.37–0.69)	3.00E-05
	PC aa C34:2	0.45 (0.31–0.63)	7.00E-06
	Hexose	0.05 (0.02–0.11)	2.00E-14

### Validation of selected metabolites in the replication set

**Fig 1:**
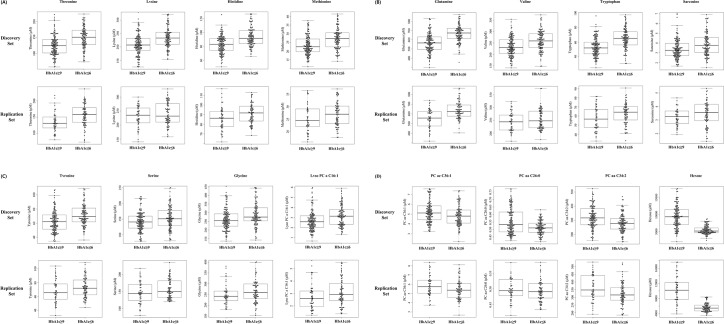
The dot- and box-plot of the concentration of 16 metabolites (A-D) which were selected having different concentration between normal (HbA1c ≤6%) and high (HbA1c ≥9%) level of HbA1c group in both discovery and replication study. Graph was made by using R package (STATS v3.6.1). Y-axis indicates the concentration of metabolites as micromoles (μM).

To validate whether the 22 metabolites identified in the discovery set are significantly associated with the level of HbA1c, metabolomic profiling was performed using a replication set. Following the same analysis procedures used for discovery set, but using age as the only covariate, 21 metabolites, including 16 metabolites identified in the discovery set, were selected as significantly differentially expressed metabolites depending on the level of HbA1c (Table 3). Fifteen metabolites showed higher concentrations and six metabolites showed lower concentrations in the normal group than in the group with high HbA1c. Twelve metabolites (i.e., threonine, lysine, histidine, methionine, glutamine, valine, tryptophan, sarcosine, tyrosine, serine, glycine, and lysoPC a C16:1) showed significantly higher concentrations in the group with a normal level of HbA1c in both the discovery and replication sets. Four metabolites (i.e., PC ae C36:1, PC aa C26:0, PC aa C34:2, and hexose) showed significantly lower concentrations in the group with a normal level of HbA1c in both the discovery and replication sets (Fig 1).

**Table 3:** Identification of metabolites with different concentrations depending on the level of HbA1c by a multivariate logistic regression analysis in the replication set.

Group	Metabolites	Odds Ratio (CI)	FDR-Corrected P-value
High Concentrations in the Normal HbA1c Group	Threonine*	3.77 (2.38–6.32)	9.00E-08
	Lysine*	3.22 (2.02–5.46)	4.00E-06
	Histidine*	2.97 (1.91–4.91)	6.00E-06
	Methionine*	2.94 (1.90–4.81)	5.00E-06
	Glutamine*	2.87 (1.84–4.71)	1.00E-05
	Valine*	2.84 (1.86–4.57)	5.00E-06
	Tryptophan*	2.83 (1.85–4.54)	5.00E-06
	Sarcosine*	2.62 (1.64–4.47)	2.00E-04
	Proline	2.40 (1.57–3.81)	1.00E-04
	Tyrosine*	2.32 (1.56–3.59)	7.00E-05
	Serine*	2.13 (1.45–3.27)	3.00E-04
	Glycine*	2.04 (1.39–3.09)	4.00E-04
	Phenylalanine	1.90 (1.28–2.92)	2.00E-03
	Ornithine	1.89 (1.28–2.94)	3.00E-03
	lysoPC a C16:1*	1.79 (1.24–2.65)	2.00E-03
Low Concentrations in the Normal HbA1c Group	PC ae C36:2	0.54 (0.36–0.78)	2.00E-03
	C4	0.54 (0.34–0.81)	6.00E-03
	PC ae C36:1*	0.53 (0.35–0.79)	2.00E-03
	PC aa C26:0*	0.46 (0.28–0.72)	1.00E-03
	PC aa C34:2*	0.44 (0.28–0.68)	3.00E-04
	Hexose*	0.24 (0.08–0.55)	5.00E-03

*Selected metabolites in both the discovery and replication sets

## Discussion

Using a targeted metabolomics approach, metabolites associated with the level of HbA1c in the serum of patients with diabetes were discovered and validated. Twenty-two metabolites were identified in the discovery set and sixteen of these metabolites were validated in the replication set. The concentrations of twelve metabolites, including eleven amino acids and one lysoPC (lysoPC a C16:1), were higher and those of four metabolites, including three PCs (PC ae C36:1, PC aa C26:0, and PC aa C34:2) and hexose, were lower in the group with normal levels of HbA1c than in the group with high level of HbA1c. Therefore, these metabolites might be linked to the concentration of HbA1c in the serum of T2D patients.

In this study, the concentrations of many amino acids were significantly higher in the group with normal levels of HbA1c than in the group with high levels of HbA1c. This result is consistent with previous results in cases of certain amino acids such as glycine and glutamine [6, 16, 17]. It was reported that glucogenic amino acids such as glycine, aspartate, serine, glutamine and histidine were in low concentrations in rats with diabetes [18]. In a human metabolome study, glycine was lower in patients with T2D and prediabetes than those with normal glucose tolerance. This suggests that glycine levels may be inversely proportional to gluconeogenesis and glucose concentrations [4–6]. Furthermore, the concentration of glutamine was increased in group with normal HbA1c levels, which is in accordance with previous study [16]. It was also reported that glutamine is associated with insulin sensitivity and reduced diabetes risk. The strong association of insulin-resistance traits with glutamine was shown in both cohorts in that study. However, the association of high glutamine-glutamate ratio with lower risk of incident diabetes was shown in one cohort but not in another. Additionally, they also showed that administration of glutamine in mice resulted in increased glucose tolerance and decreased blood pressure [19].

Levels of BCAAs (branched-chain amino acids; isoleucine, leucine, and valine) in the blood are elevated in patients with impaired fasting glucose and T2D [20]. However, in a population-based cohort study, high intake of BCAAs was associated with a decrease in diabetes risk, although the mechanism and regulation of amino acid uptake and concentration in serum was not defined completely [21]. In our study, other than valine, isoleucine and leucine did not show significantly different concentrations between groups with high and low levels of HbA1c. However, the concentration of valine was higher in the group with a normal level of HbA1c than in the group with a high level of HbA1c.

Several studies have identified PC metabolites associated with diabetes progression. In a longitudinal study, low levels of acyl-alkyl PCs (PC ae) and high levels of triacylglycerols and diacyl-phospholipids were observed during progression to T2D [22]. Several reports have shown that the concentration of lysoPC a 18:2 is reduced in T2D [4–6]. However, the concentration of the metabolite lysoPC a 18:2 did not differ between groups with normal and high levels of HbA1c in our study, even though it is included in the AbsoluteIDQ^®^ p180 Kit that was used in this study. Based on previous results and those of our study, lysoPC a 18:2 may be involved in the progression of diabetes but not in the reverse process from high to normal levels of HbA1c. Furthermore, the concentration of one acyl lysoPC (lysoPC a C16:1) was higher but the concentrations of two PCs (PC aa C34:2, PC ae C36:1) were lower in the group with a normal level of HbA1c than in the group with a high level of HbA1c in our study. Based on these results, PC concentrations differed between the two groups (with normal and high levels of HbA1c). These PCs might be good candidates to elucidate the mechanisms of lowered glucose level in blood.

Although it is not easy to elucidate the pathway of amino acids catabolism and the mechanism of maintaining the level of amino acids in serum, a correlation between the intake and serum level of amino acids may exist. Certain amino acids may not necessarily regulate HbA1c in diabetic patients. However, considering previous reports showing that amino acid supplementation decreases HbA1c and glucose levels in patients with diabetes, amino acids identified in this study could play important roles in regulating HbA1c levels in T2D patients [23, 24]. Further studies, such as validation experiments using mouse models and lipidomic analyses using a non-targeted metabolomics approach, will be helpful to identify more metabolites related to the T2D condition and to elucidate the mechanism underlying the lowering of HbA1c to normal levels. Comprehensive metabolomic profiles could provide metabolite signatures associated with HbA1c levels and will be beneficial to elucidate the mechanism by which HbA1c levels can be regulated in diabetic patients.

## Supporting information

S1 Table Characteristics of 122 selected metabolites. (XLSX)
